# Supplementary material for: Fetal biometry and amniotic fluid volume assessment end-to-end automation using Deep Learning
Source: Nat Commun. 2023 Nov 3;14:7047. doi: 10.1038/s41467-023-42438-5 (PMC10624828; doi:10.1038/s41467-023-42438-5)
Supplement: Supplementary file 1 — Supplementary Information [file 41467_2023_42438_MOESM1_ESM.pdf]

Fetal biometry and amniotic fluid volume assessment end-to-end automation using Deep Learning

Supplementary information

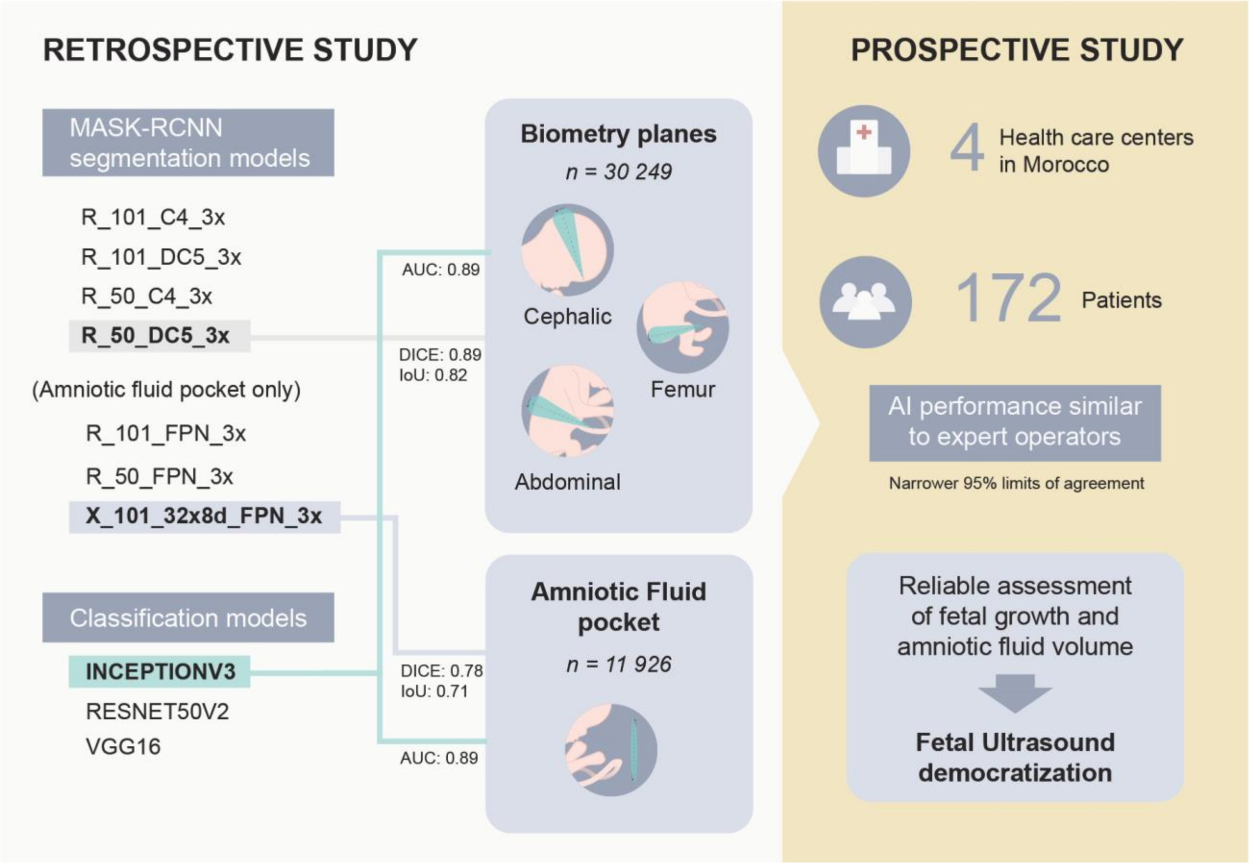

Supplementary Figure 1: Graphical Abstract. We conducted the first phase of development and testing of our approach on a retrospective dataset comprising 42,175 fetal ultrasound images. We then validated our approach prospectively on 172 patients in 4 healthcare centers in Morocco, showing narrower limits of agreement than human operators of all the studied parameters. Helena Pinheiro https://www.hpinheiro.com/ created this illustration

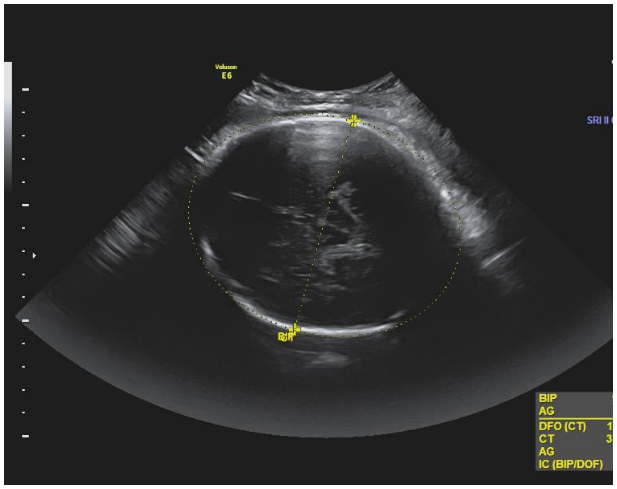

(1) Original image

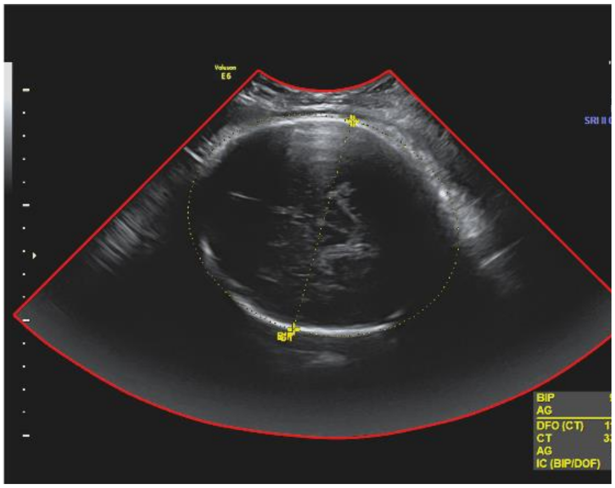

(2) ROI detection

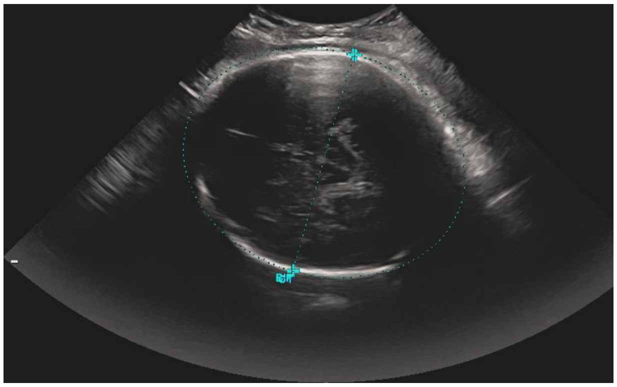

(3) Calipers and dots detection

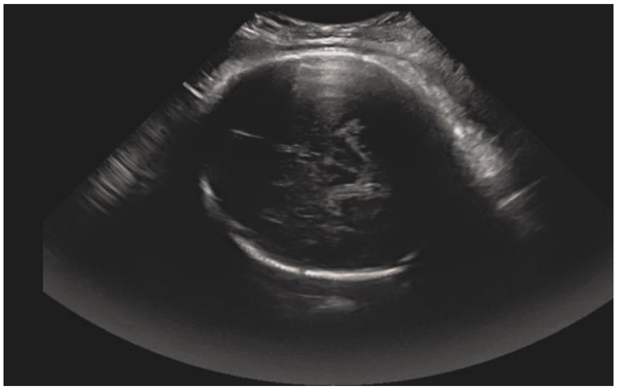

(4) Cleaned image

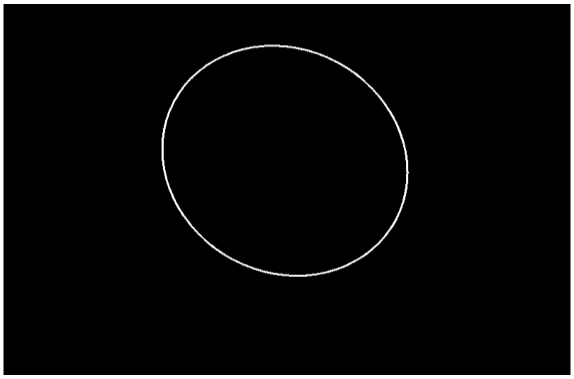

(5) Mask extraction (validated by annotator)

Brain Standard Plane
BPD: 9,40cm
HC: 33,65cm

(6) Standard plan and biometric measurement recognition using OCR (validated by annotator)

Supplementary Figure 2: Preprocessing steps for generating ground truth masks and standard plan and biometric measurement recognition. (1) original image of a brain plan with pixelated yellow calipers, dotted circumference, and biometric measurements (2) region of interest

detection (3) Calipers and dots detection within the region of interest (4) cleaned image using inpainting techniques (5) mask extraction and validation by the annotators and (6) Standard plan and biometric measurement recognition using OCR techniques.

Reference	Task	Data	Measurement	Method	Performance	Main Contribution
Baumgartner et al. [42] (2017)	2D Standard Plan Detection	140,827 2D images and 2,438 videos	13 Standard Planes	SonoNet (Adapted VGG16)	Mean F1 = 0.80	Detection of 13 fetal standard planes.
Cai et al. [43] (2020)	2D Standard Plan Detection	280 2D videos (3-7)s	Abdomen, Femur and Brain standard planes	biCLSTM with Temporal Attention	Mean F1 = 0.85	Better performance in the classification of three standard planes
Zeng et al. [15] (2021)	2D Fetal biometry	999 2D images (HC18)	HC	V-Net with attention	Dice = 0.98<br>MAE = 1.77mm	Best segmentation performance of HC on a public dataset.
Moccia et al. [26] (2021)	2D Fetal Biometry	999 2D images (HC18)	HC	Adapted Mask-RCNN	Dice = 0.98<br>MAE = 1.95mm	Best segmentation performance of HC on a public dataset.
Plotka et al. [14] (2022)	2D Fetal Biometry	274,275 2D images from videos	HC, AC, FL, GA, EFW	FUVAI (U-Net + ConvLSTM)	Dice = 0.96<br>MAE = 2.5mm<br>GA MAE = 0.35d<br>EFW MAE = 25g	Best segmentation performance of FB, validated on a large dataset.
Pokaprakarnet al. [18] (2022)	2D Fetal Biometry	147,855 2D images (FAMLI)	GA	Deep learning model	GA MAE = 3.9d	end-to-end GA estimation.
Gomes et al. [20] (2022)	2D Fetal Biometry	147,855 2D images (FAMLI)	GA	Deep learning model	GA MAE = -1.4d	end-to-end GA estimation in a Mobile setting.
Cho et al. [28] (2021)	2D Amniotic Fluid	310 2D images	SDP	AF-Net (Adapted U-Net)	Dice = 0.87<br>SDP MAE = 2.66cm	Best segmentation performance, yet validated on a very small dataset.
**Our Method**	2D Standard Plan Detection, 2D Fetal Biometry and Quality Criteria	30,249 2D images	HC, BPD, AC, FL, GA, EFW	Mask-RCNN INCEPTIONV3	Mean F1 = 0.73<br>Dice = 0.89<br>MAE = 6.1mm<br>GA MAE = 9.85d<br>EFW MAE = 147.18g	use of ISUOG guidelines to assess the quality of all three standard biometry planes validated prospectively on a larger dataset of African patients.
**Our Method**	2D Amniotic Fluid	11,926 2D images	SDP	Mask-RCNN INCEPTIONV3	F1 = 0.81<br>Dice = 0.78<br>SDP MAE = 1.46cm	Blind loops to measure the SDP that is validated prospectively on a larger dataset of African patients.

Supplementary Table 1: Best-performing deep learning models reported in the state-of-the-art literature for the classification and segmentation tasks to detect 2D standard planes, and assess fetal biometry or amniotic fluid volume. The two last rows present the results of our models, and the last column reports the main contributions.

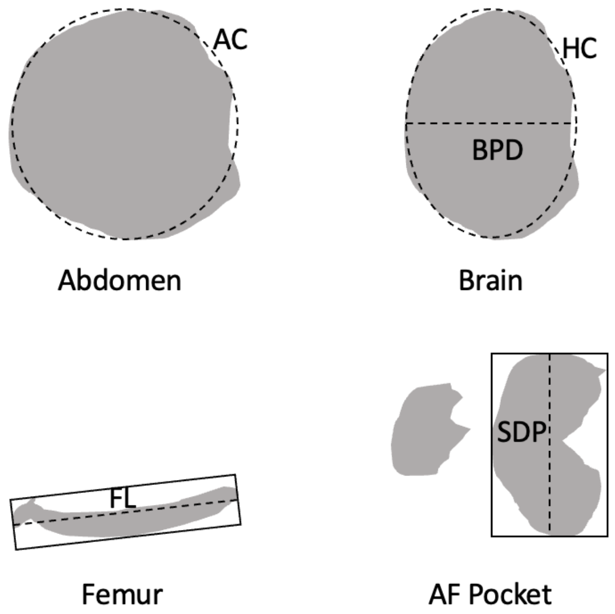

Supplementary Figure 3: Approximations of the biometric measurements. For the abdomen and brain, AC and HC are the circumferences of the ellipses approximated by first finding the contours and then using the direct least square fitting method, and BPD is the minor axis of the brain ellipse. For the femur, FL represents the measure of the major axis of the extremities bounding box, and for the AF pocket, the SDP is the measure of the vertical axis of the bounding box.

Section & Topic	No	Item	Reported on page #
**TITLE OR ABSTRACT**			
	**1**	Identification as a study of diagnostic accuracy using at least one measure of accuracy (such as sensitivity, specificity, predictive values, or AUC)	1
**ABSTRACT**			
	**2**	Structured summary of study design, methods, results, and conclusions (for specific guidance, see STARD for Abstracts)	3
**INTRODUCTION**			
	**3**	Scientific and clinical background, including the intended use and clinical role of the index test	4-5
	**4**	Study objectives and hypotheses	5
**METHODS**			

Study design	**5**	Whether data collection was planned before the index test and reference standard were performed (prospective study) or after (retrospective study)	16-17
Participants	**6**	Eligibility criteria	17
	**7**	On what basis potentially eligible participants were identified (such as symptoms, results from previous tests, inclusion in registry)	17
	**8**	Where and when potentially eligible participants were identified (setting, location and dates)	17
	**9**	Whether participants formed a consecutive, random or convenience series	17
Test methods	**10a**	Index test, in sufficient detail to allow replication	14-17
	**10b**	Reference standard, in sufficient detail to allow replication	17
	**11**	Rationale for choosing the reference standard (if alternatives exist)	NA
	**12a**	Definition of and rationale for test positivity cut-offs or result categories of the index test, distinguishing pre-specified from exploratory	16-17
	**12b**	Definition of and rationale for test positivity cut-offs or result categories of the reference standard, distinguishing pre-specified from exploratory	16-17
	**13a**	Whether clinical information and reference standard results were available to the performers/readers of the index test	17
	**13b**	Whether clinical information and index test results were available to the assessors of the reference standard	17
Analysis	**14**	Methods for estimating or comparing measures of diagnostic accuracy	17-18
	**15**	How indeterminate index test or reference standard results were handled	9
	**16**	How missing data on the index test and reference standard were handled	9
	**17**	Any analyses of variability in diagnostic accuracy, distinguishing pre-specified from exploratory	10
	**18**	Intended sample size and how it was determined	16-17
**RESULTS**			
Participants	**19**	Flow of participants, using a diagram	33
	**20**	Baseline demographic and clinical characteristics of participants	9
	**21a**	Distribution of severity of disease in those with the target condition	NA
	**21b**	Distribution of alternative diagnoses in those without the target condition	NA
	**22**	Time interval and any clinical interventions between index test and reference standard	17
Test results	**23**	Cross tabulation of the index test results (or their distribution) by the results of the reference standard	27
	**24**	Estimates of diagnostic accuracy and their precision (such as 95% confidence intervals)	9-10
	**25**	Any adverse events from performing the index test or the reference standard	11
**DISCUSSION**			
	**26**	Study limitations, including sources of potential bias, statistical uncertainty, and generalisability	13-14
	**27**	Implications for practice, including the intended use and clinical role of the index test	13
**OTHER INFORMATION**			
	**28**	Registration number and name of registry	18
	**29**	Where the full study protocol can be accessed	18
	**30**	Sources of funding and other support; role of funders	26

STARD 2015 Checklist

AIM

STARD stands for "Standards for Reporting Diagnostic accuracy studies". This list of items was developed to contribute to the completeness and transparency of reporting of diagnostic accuracy studies. Authors can use the list to write informative study reports. Editors and peer-reviewers can use it to evaluate whether the information has been included in manuscripts submitted for publication.

EXPLANATION

A **diagnostic accuracy study** evaluates the ability of one or more medical tests to correctly classify study participants as having a **target condition.** This can be a disease, a disease stage, response or benefit from therapy, or an event or condition in the future. A medical test can be an imaging procedure, a laboratory test, elements from history and physical examination, a combination of these, or any other method for collecting information about the current health status of a patient.

The test whose accuracy is evaluated is called **index test.** A study can evaluate the accuracy of one or more index tests. Evaluating the ability of a medical test to correctly classify patients is typically done by comparing the distribution of the index test results with those of the **reference standard**. The reference standard is the best available method for establishing the presence or absence of the target condition. An accuracy study can rely on one or more reference standards.

If test results are categorized as either positive or negative, the cross tabulation of the index test results against those of the reference standard can be used to estimate the **sensitivity** of the index test (the proportion of participants *with* the target condition who have a positive index test), and its **specificity** (the proportion *without* the target condition who have a negative index test). From this cross tabulation (sometimes referred to as the contingency or "2x2" table), several other accuracy statistics can be estimated, such as the positive and negative **predictive values** of the test. Confidence intervals around estimates of accuracy can then be calculated to quantify the statistical **precision** of the measurements.

If the index test results can take more than two values, categorization of test results as positive or negative requires a **test positivity cut-off**. When multiple such cut-offs can be defined, authors can report a receiver operating characteristic (ROC) curve which graphically represents the combination of sensitivity and specificity for each possible test positivity cut-off. The **area under the ROC curve** informs in a single numerical value about the overall diagnostic accuracy of the index test.

The **intended use** of a medical test can be diagnosis, screening, staging, monitoring, surveillance, prediction or prognosis. The **clinical role** of a test explains its position relative to existing tests in the clinical pathway. A replacement test, for example, replaces an existing test. A triage test is used before an existing test; an add-on test is used after an existing test.

Besides diagnostic accuracy, several other outcomes and statistics may be relevant in the evaluation of medical tests. Medical tests can also be used to classify patients for purposes other than diagnosis, such as staging or prognosis. The STARD list was not explicitly developed for these other outcomes, statistics, and study types, although most STARD items would still apply.

DEVELOPMENT

This STARD list was released in 2015. The 30 items were identified by an international expert group of methodologists, researchers, and editors. The guiding principle in the development of STARD was to select items that, when reported, would help readers to judge the potential for bias in the study, to appraise the applicability of the study findings and the validity of conclusions and recommendations. The list represents an update of the first version, which was published in 2003.

More information can be found on http://www.equator-network.org/reporting-guidelines/stard.

Sample Size Estimation

```
remove(list=ls())
library(pwr)
setwd("~/Documents/deep_vecho")

data<-read.csv('diffall2 - diffall2.csv', head=T)

table(data$Classe)
```

```
##
##       Abdominal            Femur Transthalamique
##            3804             2729            3766
```

```
data_abd<-data[data$Classe=='Abdominal',] data_femur<-
data[data$Classe=='Femur',] data_transth<-
data[data$Classe=='Transthalamique',]

summary(data_femur$Difference.cm.)
```

```
##    Min. 1st Qu. Median    Mean 3rd Qu.    Max.
##    0.00    0.81    1.93    1.73    2.39    8.75
```

```
summary(data_abd$Difference.cm.)
```

```
##    Min. 1st Qu. Median    Mean 3rd Qu.    Max.
##    0.00    0.21    0.46    6.94    0.89 3015.45
```

```
summary(data_transth$Difference.cm.)
```

```
##      Min.  1st Qu.    Median     Mean 3rd Qu.       Max.
##     0.000    0.150     0.330    4.359    0.580  3297.950
```

```
## remove outliers

##abd


outliers_3<- boxplot(data_abd$Difference.cm.,

plot=FALSE)$out outliers<- outliers_3

x_abd<-data_abd
x_abd<- x_abd[-which(x_abd$Difference.cm. %in% outliers),]
summary(x_abd)
```

```
##      Classe           ExpertsMeasurement.cm.
ModelsMeasurement.cm. ## Length:3535            Min.   : 5.96
       Min.             : 6.14
```

```
##   Class :character    1st Qu.:17.02           1st Qu.:17.20
##   Mode :character     Median :19.10           Median :19.24
##                       Mean    :20.15          Mean    :20.21
##                       3rd Qu.:22.64           3rd Qu.:22.68
##                       Max.    :39.66          Max.    :39.86
##   Difference.cm.          DSC
##   Min.    :0.0000    Min.    :0.4690
##   1st Qu.:0.2000     1st Qu.:0.9310
##   Median :0.4100     Median :0.9476
##   Mean    :0.5355    Mean    :0.9363
##   3rd Qu.:0.7750     3rd Qu.:0.9604
##   Max.    :1.9100    Max.    :0.9856
```

```
set.seed(123)
sample1<-sample(x_abd$Difference.cm., 2000)
sd1<-sd(sample1)
diff_m<-mean(sample1)
es_abd<-diff_m/sd1
es_abd
```

```
## [1] 1.223328
```

```
sz_abd<- pwr.t.test(d=es_abd, sig.level = 0.01, power = 0.9, type = 'paired', alternative    'two.sided'
sz_abd$n
```

```
## [1] 13.39334
```

```
##
m<-mean(x_abd$DSC)
sd<-sd(x_abd$DSC)
dsc_abd<- pwr.t.test(d=(m-0.925)/sd, sig.level = 0.05, power = 0.8, type = 'one.sample', alternative =

dsc_abd$n
```

```
## [1] 121.1084
```

```
##femur


outliers_3<- boxplot(data_femur$Difference.cm., plot=FALSE)$out

outliers<- outliers_3

x_femur<-data_femur
x_femur<- x_femur[-which(x_femur$Difference.cm. %in% outliers),]
summary(x_femur)
```

```
##     Classe           ExpertsMeasurement.cm.  ModelsMeasurement.cm.  ##
Length:2725 Min.  : 0.970 Min.  : 1.215
##   Class :character    1st Qu.: 3.780        1st Qu.: 4.945 ##
Mode    :character     Median  : 4.260         Median  : 6.050  ##
                          Mean   : 4.445        Mean   : 6.101
##                        3rd Qu.: 5.010         3rd Qu.: 7.060
##                        Max.    :11.800       Max.    :11.580 ##
## Difference.cm.       DSC
## Min.   :0.000   Min.   : NA
```

```
## 1st Qu.:0.800  1st Qu.: NA
##  Median :1.930  Median : NA ##
Mean  :1.723   Mean   :NaN ##  3rd
Qu.:2.390   3rd Qu.:  NA  ##   Max.
:4.700   Max.    : NA ##     NA's
:2725
```

```
####
set.seed(1234)
sample1<-sample(x_femur$Difference.cm., 2000)
sd1<-sample(1)
diff_m<-mean(sample1)
es_abd<-diff_m/sd1
es_abd
```

```
## [1] 1.706055
```

```
sz_femur<- pwr.t.test(d=es_abd, sig.level = 0.01, power = 0.9, type = 'paired', alternative = 'two.side
sz_femur$n
```

```
## [1] 8.589127
```

```
#####


##transth


outliers_3<- boxplot(data_transth$Difference.cm., plot=FALSE)$out

outliers<- outliers_3

x_transth<-data_transth
x_transth<- x_transth[-which(x_transth$Difference.cm. %in% outliers),]
summary(x_transth)
```

```
##     Classe           ExpertsMeasurement.cm.  ModelsMeasurement.cm.
##  Length:3597         Min.   : 6.70           Min.   : 7.02
##  Class :character    1st Qu.:19.46           1st Qu.:19.53
##  Mode  :character    Median :21.56           Median :21.74
##                      Mean   :22.59           Mean   :22.72
##                      3rd Qu.:25.59           3rd Qu.:25.81
##                      Max.   :36.86           Max.   :36.63
##  Difference.cm.          DSC
##  Min.   :0.0000    Min.   :0.1901
##  1st Qu.:0.1400    1st Qu.:0.9600
##  Median :0.3100    Median :0.9682
##  Mean   :0.3733    Mean   :0.9660
##  3rd Qu.:0.5400    3rd Qu.:0.9747
##  Max.   :1.2200    Max.   :0.9905
```

```
###
set.seed(1234567)
sample1<-sample(x_transth$Difference.cm., 2000)
sd1<-sample(1)
diff_m<-mean(sample1)
```

```
es_abd<-
diff_m/sd1
```

```
## [1] 0.37323
```

```
sz_transth<- pwr.t.test(d=es_abd, sig.level = 0.01, power = 0.9, type = 'paired', alternative = 'two.si
sz_transth$n
```

```
## [1] 110.1528
```

```
m<-mean(x_transth$DSC)
sd<-sd(x_transth$DSC)
dsc_transth<- pwr.t.test(d=(m-0.96)/sd, sig.level = 0.05, power = 0.8, type = 'one.sample', alternative

dsc_transth$n
```

```
## [1] 57.37911
```

Final sample size

```
max(sz_abd$n, sz_femur$n, sz_transth$n, dsc_abd$n, dsc_transth$n )
```

```
## [1] 121.1084
```
