# Supplementary material for: Fetal biometry and amniotic fluid volume assessment end-to-end automation using Deep Learning
Source: Nat Commun. 2023 Nov 3;14:7047. doi: 10.1038/s41467-023-42438-5 (PMC10624828; doi:10.1038/s41467-023-42438-5)
Supplement: Supplementary file 3 — Description of Additional Supplementary Files [file 41467_2023_42438_MOESM3_ESM.pdf]

File name: Supplementary Data 1

Description: Dataset containing the predicted and measured biometric parameters for each of the included participants
