# Supplementary material for: Fetal biometry and amniotic fluid volume assessment end-to-end automation using Deep Learning
Source: Nat Commun. 2023 Nov 3;14:7047. doi: 10.1038/s41467-023-42438-5 (PMC10624828; doi:10.1038/s41467-023-42438-5)
Supplement: Supplementary file 5 — Reporting Summary [file 41467_2023_42438_MOESM5_ESM.pdf]

nature portfolio

Corresponding author(s): Saad Slimani

Last updated by author(s): Jul 20, 2023

Reporting Summary

Nature Portfolio wishes to improve the reproducibility of the work that we publish. This form provides structure for consistency and transparency in reporting. For further information on Nature Portfolio policies, see our Editorial Policies and the Editorial Policy Checklist.

Statistics

For all statistical analyses, confirm that the following items are present in the figure legend, table legend, main text, or Methods section.

n/a	Confirmed	
☐	☒	The exact sample size (*n*) for each experimental group/condition, given as a discrete number and unit of measurement
☐	☒	A statement on whether measurements were taken from distinct samples or whether the same sample was measured repeatedly
☒	☐	The statistical test(s) used AND whether they are one- or two-sided<br>*Only common tests should be described solely by name; describe more complex techniques in the Methods section.*
☒	☐	A description of all covariates tested
☐	☒	A description of any assumptions or corrections, such as tests of normality and adjustment for multiple comparisons
☐	☒	A full description of the statistical parameters including central tendency (e.g. means) or other basic estimates (e.g. regression coefficient) AND variation (e.g. standard deviation) or associated estimates of uncertainty (e.g. confidence intervals)
☒	☐	For null hypothesis testing, the test statistic (e.g. *F*, *t*, *r*) with confidence intervals, effect sizes, degrees of freedom and *P* value noted<br>*Give P values as exact values whenever suitable.*
☒	☐	For Bayesian analysis, information on the choice of priors and Markov chain Monte Carlo settings
☐	☒	For hierarchical and complex designs, identification of the appropriate level for tests and full reporting of outcomes
☒	☐	Estimates of effect sizes (e.g. Cohen's *d*, Pearson's *r*), indicating how they were calculated

Our web collection on statistics for biologists contains articles on many of the points above.

Software and code

Policy information about availability of computer code

Data collection	A web application for data collection developed internally and hosted on Microsoft Azure cloud. Both Tensorflow and Pytorch python framework were used for model training, testing and validation.
Data analysis	Tensorflow (version 2.0)<br>Keras (2.3.1)<br>Pytorch (version 1.10)<br>R package 'Metrics' (version 0.1.4) of R software (R version 4.2.1)<br>PairedData' (version 1.1.1) R package<br>Package 'merTools' (version 0.5.2)<br>Plots were generated using the package ggplot2 (version 3.3.6)

For manuscripts utilizing custom algorithms or software that are central to the research but not yet described in published literature, software must be made available to editors and reviewers. We strongly encourage code deposition in a community repository (e.g. GitHub). See the Nature Portfolio guidelines for submitting code & software for further information.

Data

Policy information about availability of data

All manuscripts must include a data availability statement. This statement should provide the following information, where applicable:

- Accession codes, unique identifiers, or web links for publicly available datasets
- A description of any restrictions on data availability
- For clinical datasets or third party data, please ensure that the statement adheres to our policy

Source data are provided with this paper.
Part of the de-identified fetal ultrasound data used in this study comes from a publicly available dataset on Zenodo published by Burgos-Artizzu, X. P. et al.17 available at https://zenodo.org/record/3904280
The rest of the de-identified fetal ultrasound data is not made publicly available for ethical and legal considerations.
The pre-trained models used in this study are available publicly. Their fine-tuned weights can be made available for research and reproducibility purposes upon request to the corresponding author at saad.slimani@deepecho.io within 14 days, subject to a data use agreement for non-commercial use.

Human research participants

Policy information about studies involving human research participants and Sex and Gender in Research.

Reporting on sex and gender	All of the study participants were of female sex, the gender identity of each of the participants was not recorded during the study.
Population characteristics	172 patients with singleton pregnancies were included in our prospective study. Multiple pregnancies were not an exclusion criterion, and patients were included even in the case of partially complete examinations The patients were all at least 18 years old
Recruitment	Patients were recruited in a consecutive fashion. Participants who presented themselves for a fetal ultrasound scan int the participating centers were offered to be part of the clinical study. After consent was obtained they were assessed for inclusion and exclusion criteria.
Ethics oversight	CERBO: Oujda Biomedical Research Ethics Committee

Note that full information on the approval of the study protocol must also be provided in the manuscript.

Field-specific reporting

Please select the one below that is the best fit for your research. If you are not sure, read the appropriate sections before making your selection.

☒ Life sciences ☐ Behavioural & social sciences ☐ Ecological, evolutionary & environmental sciences

For a reference copy of the document with all sections, see nature.com/documents/nr-reporting-summary-flat.pdf

Life sciences study design

All studies must disclose on these points even when the disclosure is negative.

Sample size	172 patients, initial estimated initial sample size was of 122. A detailed explanation of the sample size calculations is provided as a supplementary information file.
Data exclusions	Duplicates and patients without an image nor cine-loop available or no corresponding ground truth measurement obtained were excluded. In total, the study gathered: 142 different cine-loops containing a femoral plane; 144 containing an abdominal plane; 123 containing a cephalic plane; and 90 containing AF-pockets
Replication	The study was divided in a retrospective testing of the models using the same sites, ultrasound machines, and sonographers. And a prospective validation study in additional sites, using different ultrasound machines and with different physicians to ensure the models' generalizability. Thus one replication with a different data set from the retrospective test-training sets was performed. This replication attempt was successful as detailed in the manuscript.
Randomization	Images were randomly allocated to the training, testing and validation sets.
Blinding	Predictions of the models were unavailable to the sonographers at the time of the study.

Reporting for specific materials, systems and methods

We require information from authors about some types of materials, experimental systems and methods used in many studies. Here, indicate whether each material, system or method listed is relevant to your study. If you are not sure if a list item applies to your research, read the appropriate section before selecting a response.

Materials & experimental systems

n/a	Involved in the study
☒	☐ Antibodies
☒	☐ Eukaryotic cell lines
☒	☐ Palaeontology and archaeology
☒	☐ Animals and other organisms
☐	☒ Clinical data
☒	☐ Dual use research of concern

Methods

n/a	Involved in the study
☒	☐ ChIP-seq
☒	☐ Flow cytometry
☒	☐ MRI-based neuroimaging

Clinical data

Policy information about clinical studies

All manuscripts should comply with the ICMJE guidelines for publication of clinical research and a completed CONSORT checklist must be included with all submissions.

Clinical trial registration

NCT05059093

Study protocol

https://clinicaltrials.gov/ct2/show/NCT05059093

Data collection

Retrospecitve data was collected in two private practices in Casablanca, Morocco Ibn Rochd University Hospital departments of Radiology and Obstetrics, Casablanca, and Mohammed V University Hospital's department of obstetrics in Oujda, Moroccobetween 2015 and 2021. Not all the retrospective data was used for model training and testing.

Prospective data was collected from the same centers except one private practice in Casablanca from October 2021 to April 2022

Outcomes

The primary outcome was the models performances in fetal biometry and single deepest pocket measurements, expressed in mean absolute error, limits of agreement with the sonographers, and Intra class correlation coefficients.

The secondary outcomes were the performances of the models at detecting AFV and fetal growth abnormalities (FGR) using sensitifivity, and specificity metrics.
